# Nitrogen Chemistry and Coke Transformation of FCC Coked Catalyst during the Regeneration Process

**DOI:** 10.1038/srep27309

**Published:** 2016-06-08

**Authors:** Junjun Shi, Jianyu Guan, Dawei Guo, Jiushun Zhang, Liam John France, Lefu Wang, Xuehui Li

**Affiliations:** 1 School of Chemistry and Chemical Engineering, Pulp & Paper Engineering State Key Laboratory of China, South China University of Technology, Guangzhou 510640, China; 2 Research Institute of Petroleum Processing Sinopec, Beijing 100083, China

## Abstract

Regeneration of the coked catalyst is an important process of fluid catalytic cracking (FCC) in petroleum refining, however, this process will emit environmentally harmful gases such as nitrogen and carbon oxides. Transformation of N and C containing compounds in industrial FCC coke under thermal decomposition was investigated via TPD and TPO to examine the evolved gaseous species and TGA, NMR and XPS to analyse the residual coke fraction. Two distinct regions of gas evolution are observed during TPD for the first time, and they arise from decomposition of aliphatic carbons and aromatic carbons. Three types of N species, pyrrolic N, pyridinic N and quaternary N are identified in the FCC coke, the former one is unstable and tends to be decomposed into pyridinic and quaternary N. Mechanisms of NO, CO and CO_2_ evolution during TPD are proposed and lattice oxygen is suggested to be an important oxygen resource. Regeneration process indicates that coke-C tends to preferentially oxidise compared with coke-N. Hence, new technology for promoting nitrogen-containing compounds conversion will benefit the *in-situ* reduction of NO by CO during FCC regeneration.

Fluid catalytic cracking (FCC) is one of the most important processes in petroleum refining for converting high-boiling hydrocarbon fractions into valuable products, particularly gasoline and light cycle oil^1^. During the cracking process, a fraction of feedstock deposits on the acid sites of the FCC catalyst, and transforms into coke via a series of polymerisation and dehydrogenation reactions, which is reversible via oxidation regeneration^2^,^3^. However, FCC catalyst deactivation also occurs irreversibly, including the deposition of metal species (Ni, V, Fe, etc.)^1^,^4^, and dealumination of the zeolite catalyst component^4^,^5^. This is effectively attenuated by continual removal of degraded catalyst and addition of fresh catalyst, resulting in an equilibrium catalyst mixture. In order to regenerate the coked catalyst, combustion is generally adopted to burn off the coke, but it results in NO*x* emission simultaneously due to the oxidation of organic nitrogen compounds enriched in coke^6^. It has been reported that NO*x* emission from FCC regeneration accounts for 50% of the refinery total NO*x* emissions, and the primary component is NO (>95%) with small amounts of N_2_O and NO_2_^7^,^8^.

Typically, there are three kinds of complex chemical reactions contributing to combustion NO*x* emission: fuel NO*x*, thermal NO*x* and prompt NO*x*. Fuel NO*x* is derived from the oxidation of nitrogen compounds in fuel, and the others are generated through the oxidation of N_2_ (thermal NO*x*) or the reaction between N_2_ and hydrocarbon radicals (prompt NO*x*). Prompt NO*x* is generally ignored, as it constitutes the smallest source of NO*x*^9^. Thermal NO*x* can only be formed significantly above 1760 °C in the presence of excess O_2_ (much higher than FCC regeneration temperature)^7^,^10^, hence, only trace levels of NO*x* (less than 10 ppm) is derived from thermal NO*x* in a typical regenerator^11^. Dishman *et al.* reported that neither thermal NO*x* nor prompt NO*x* was detectable during the FCC regeneration with samples prepared from N-free precursor (isobutylene)^12^. Hence, fuel NO*x* is considered the main source of NO*x* emissions from FCC regeneration. As NO*x* contributes to numerous environmental problems (photochemical smog, acid rain, the greenhouse effect, *etc.*), NO*x* control technology is of paramount importance in FCC regeneration^1^,^5^. Generally, there are three major industrial routes to reduce NO*x* emission: pre-combustion control, combustion modification and post-combustion control^13^. Currently, post-combustion control technology plays an important role in the removal of NO*x* from flue gas: selective catalytic reduction (SCR)^14^,^15^,^16^,^17^, plasma catalysis^13^,^18^, and absorption^19^,^20^, but these techniques propose a number of challenges: large capital investment, catalyst deactivation and solid waste disposal. An alternative promising strategy is developing *in-situ* control technology, where NO*x* can be reduced *in-situ* or the generation of NO*x* can be suppressed^21^,^22^,^23^. However, such technology has not been fully developed, due to lack of understanding the NO*x* generation mechanism. Therefore, intensive investigation of the thermal behaviour and N transformation chemistry of coke species will be helpful in developing and improving *in-situ* control technology.

Previously, NO*x* formation during FCC regeneration was investigated, but the actual reaction mechanism has yet to be fully elucidated. The reported NO*x* formation routes during FCC regeneration are summarised below. Some of the nitrogen compounds in the coke are initially converted to volatile intermediates, such as HCN and NH_3_ (a)^24^. NO is believed to be a kind of secondary product derived from the oxidation of these nitrogen-containing intermediates (b), upon release into air it further oxidises to NO_2_ (c)^11^. Routes to N_2_ are suggested to occur due to the presence of CO in the regenerator where a reduction of NO by CO is feasible (d)^11^,^24^.


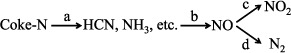


To the best of our knowledge, simulated FCC cokes prepared with nitrogen-containing precursors (like aniline, pyrrole and pyridine) have been exclusively examined^8^,^11^,^24^,^25^. However, there are significant differences in types of coke between simulated and industrial coked catalysts. The simulated catalysts exhibit only one type of coke known as catalytic coke^24^, however industrial catalysts exhibit up to three additional forms: Conradson coke, contaminate coke and catalyst-to-oil coke^4^,^26^. In the present article, an industrial coked catalyst collected from a typical FCC unit is investigated. The gaseous products from coke decomposition are monitored during temperature-programmed processes via on-line mass spectrometry and gas chromatography. The coked catalysts undergoing coke decomposition at different temperatures are analysed with NMR and XPS. Hence, exploration of the generated gaseous products, in conjunction with the evolution of the coke species, will provide further insight into the mechanism of real FCC coke transformation and yield further understanding of the formation of nitrogen and carbon based gases.

## Results and Discussion

### Thermal analysis of FCC coked catalyst

**Figure 1:**
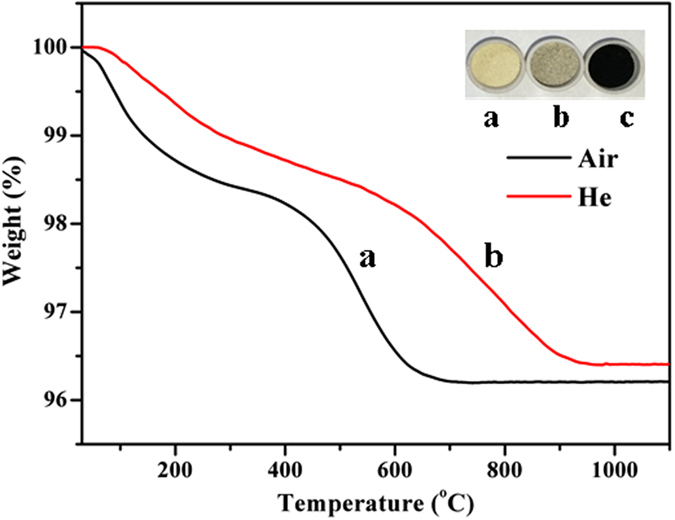
TGA of FCC coked catalyst in helium or air (**a**) the sample after testing in air, (**b**) the sample after testing in helium, (**c**) the original sample.

Thermo-gravimetric analysis (TGA) was conducted in both helium and air. TGA results (Fig. 1) indicates a rapid weight loss before 300 °C, followed by a gradual weight loss, finally the sample weight declines sharply and remains at a constant weight thereafter. The first weight loss is due to water desorption and the second is attributed to desorption (in helium)/oxidation (in air) of volatile compounds, which is due to a small quantity of steam and light hydrocarbons are retained in the pores and cavities of the industrial catalyst after stripping^4^. The last weight loss is derived from the decomposition/oxidation of stable coke compounds. Generally, FCC coke can be divided into soft (chloroform-soluble) coke and hard (chloroform-insoluble) coke, the former is primarily composed of small aliphatic molecules, and the latter is mainly attributed to stable aromatic hydrocarbons^27^,^28^. According to TGA results, the second weight loss is due to soft coke and the third weight loss is attributed to hard coke.

TGA results also show that the coke transforms much faster in air than in helium. In air, organic compounds in the coked catalyst are oxidised completely with 3.79% weight loss (Fig. 1a), while 3.59% weight loss is obtained in helium (Fig. 1b). The initial coked catalyst is black (Fig. 1c) and it turns grey after being subjected to the TGA test conducted in helium (Fig. 1b), indicating a small amount of residual carbon on the surface. For the sample tested in air, it is yellow due to the complete removal of carbon compounds by oxidation (Fig. 1a).

### Temperature programmed decomposition of FCC coked catalyst

**Figure 2:**
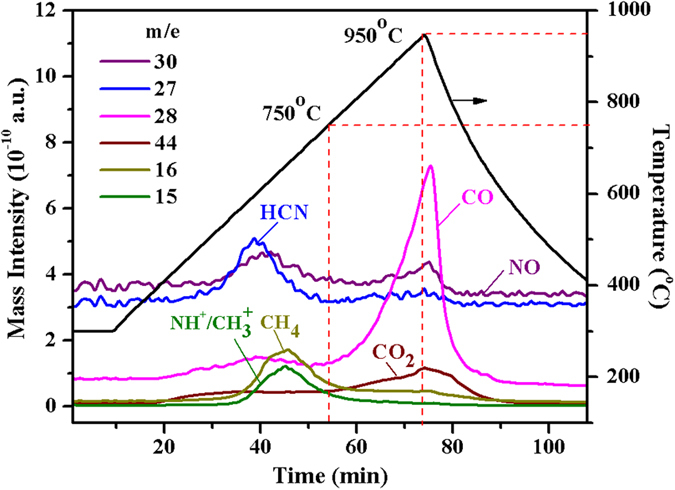
Gaseous species evolved during TPD at a uniform heating rate (*P* = 0.25 MPa; 100 mL min^−1^ of He, from 300 to 950 °C with 10 °C min^−1^. *Signals of *m*/*e* = 27 (HCN) and *m/e* = 30 (NO) have been magnified 100 times).

TPD experiments were undertaken to mimic conditions observed in TGA experiments. Two distinct regions of gas evolution are observed around 600 and 950 °C (Fig. 2), indicating the presence of two different coke types. Previous studies had shown one region occurring around 750–800 °C, attributing to a single coke type^24^. A series of experiments were conducted on coke generated from model nitrogen compounds, which resulted in the formation of catalytic coke^8^,^11^,^24^,^25^. However, coke produced from oil feedstock is somewhat more complicated, containing up to three additional forms; Conradson, contaminant and catalyst-to-oil cokes^4^,^26^.

TPD results coincide well with the TGA results (Fig. 1), which indicates that the FCC coke is constituted by two carbonic species with different thermal decomposition behaviours. NH^+^/CH_3_^+^ (*m/e* = 15), HCN (*m/e* = 27) and CO_2_ (*m/e* = 44) were detected simultaneously in the emitted gases (Fig. 2), which were observed by Barth *et al.* previously^24^. Signals of *m/e* = 30 and *m/e* = 28, representing NO and CO, were also monitored in the present study (although *m/e* of N_2_ is also 28, no N_2_ was detectable via GC analysis during TPD, due to the low level of generated N_2_, which is below the detection limit of the GC). Significantly higher content of carbon (1.59%) than nitrogen (220 ppm) in the initial coke (Table S1) results in much stronger mass intensity of carbon-containing species (CH_4_ and CO_2_, 10^−10^) than nitrogen-containing compounds (HCN and NO, 10^−12^).

The first region of gas evolution is derived from the decomposition of less stable coke, which is attributed to soft coke^27^ and decomposes at around 600 °C, generating HCN, NO, CH_4_, *etc.* (Fig. 2). The second region is attributed to the reaction of hard coke–aromatic compounds and coke deposited in the deep pores of the catalyst^28^, generating CO, NO and CO_2_ at an elevated temperature around 950 °C (Fig. 2). It is believed that NH_3_ and HCN are generated from the decomposition of nitrogen compounds in coke and are considered to be important intermediates for NO formation during FCC regeneration^11^,^24^. CH_4_ is supposed to be generated from volatile and unstable compounds retained in the coke, such as aliphatic molecules, aliphatic side chains attached to aromatics and compositions enriched with hydrogen^25^.

**Figure 3:**
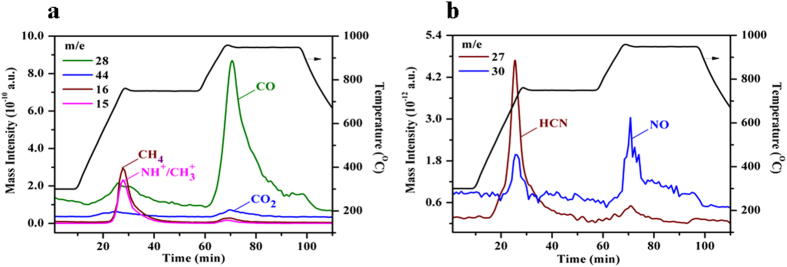
Gaseous species evolved during a four-stage TPD (**a**) carbon compounds, (**b**) nitrogen compounds; *P* = 0.25 MPa; 100 mL min^−1^ of He; from 300 to 750 °C with 25 °C min^−1^ and held for 30 min at 750 °C, then from 750 to 950 °C with 20 °C min^−1^ and finally held for 30 min at 950 °C.

To get a deep understanding of the coke decomposition, a four-stage TPD experiment was designed. The results demonstrate clearly that all gaseous products have stronger and sharper signals as a function of time (Fig. 3). Significant oxidation product formation is observed in the O_2_-free atmosphere, indicating that oxygen is derived from the coked FCC catalyst. Generally, these products are derived from three kinds of oxygen species present on the coked FCC catalyst: i) oxygen-containing compounds in the coke, ii) hydroxyl groups in the zeolite base of catalyst, iii) lattice oxygen atoms in the catalyst. Kapteijn *et al.* reported that pyridone was detected in model coke (although its content was very low), and demonstrated that CO was generated from the decomposition of pyridone when the coke was treated in an inert atmosphere at 800 °C^29^. Hydroxyl groups exist in the zeolitic component of the FCC catalyst^4^, hence, oxidation may occur between hydroxyl groups and absorbed aromatics or nitrogen-containing compounds. In isotopic exchange experiments, it is found that oxygen in ceria is mobile and can readily participate in oxidation reactions^30^,^31^, the zeolite alone makes a minor contribution to the available oxygen supply, unless it is modified by metal oxides^31^,^32^,^33^. It is further demonstrated that the combination of metal oxides and zeolite results in improved oxygen mobility within both components^31^,^32^. The catalyst employed in this study utilises zeolitic and rare earth components (primarily ceria as indicated in Table S1) in the composition. Hence, it is perceived that oxidation under TPD conditions occurs in a similar manner to that described above.

The reduction of NO by CO is considered feasible from the perspective of thermodynamics (NO + CO → 1/2 N_2_ + CO_2_, 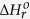 (298 K) = −373 kJ mol^−1^). According to the thermodynamic data, the Gibbs free energy (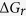) of the reaction NO + CO → 1/2 N_2_ + CO_2_ at different temperatures was calculated (Table 1, detail calculation was presented in supplementary material). The results state that the reaction is spontaneous in the temperature range employed in this study (25–950 °C). NO-TPD experiments and CO-NO reaction have been investigated^24^,^34^, it is demonstrated that NO can be readily reduced by CO to yield N_2_ and CO_2_. Catalysts for promoting this reaction during FCC regeneration have been studied^21^,^35^.

### Transformation of carbon-containing compounds in FCC coked catalyst

**Figure 4:**
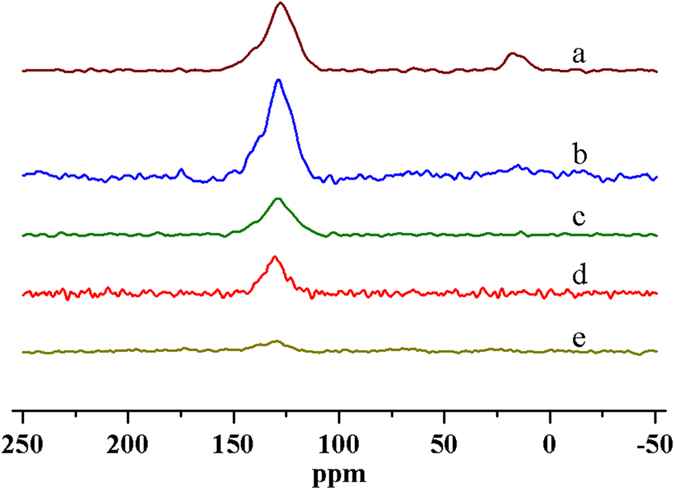
NMR spectra of FCC coked catalyst (**a**) original sample; (**b**) sample collected at the end of stage I; (**c**) sample collected at the end of stage II; (**d**) sample collected at the end of stage III; (**e**) sample collected at the end of stage IV.

To understand coke transformation during the thermal decomposition, industrial FCC coked catalysts subjected to different TPD stages were characterised by ^13^C CP-MAS NMR (carbon-13 cross-polarisation magic-angle spinning nuclear magnetic resonance). Both aromatic and aliphatic carbons are clearly identified in the initial coked catalyst, with signals centered around 130 and 20 ppm, respectively (Fig. 4a). As reported previously, typical FCC coke is dominated by aromatic carbon and a small fraction of aliphatic carbon^25^,^27^. The aliphatic species in FCC coke are primarily derived from alkyl groups attached to aromatic rings and hydrocarbons entrained in the catalyst pores (like catalyst-to-oil coke which are not removed by stripping)^4^,^25^. For samples collected at different TPD stages (Fig. 2), the signal attributed to aliphatic coke initially weakens and disappears completely after the second stage of TPD (750 °C for 30 min). This illustrates that the aliphatic coke is less stable and more readily decomposed than the aromatic coke^36^. The signal intensity of aromatic carbon barely weakens after the first stage (Fig. 4a,b), but declines at high temperature especially at the last two stages (Fig. 4d,e). Such experiments while informative are not quantitative, due to the reliance of the CP method on proton concentration. However, some general trends can be determined, considering additional evidence from TGA (Fig. 1) and TPD (Figs 2, 3). Dealkylation of coke molecules mainly takes place in earlier stages of the TPD procedure, and the reaction of aromatic coke primarily occurs at higher temperature in the later stages.

### Transformation of nitrogen-containing compounds in FCC coked catalyst

**Figure 5:**
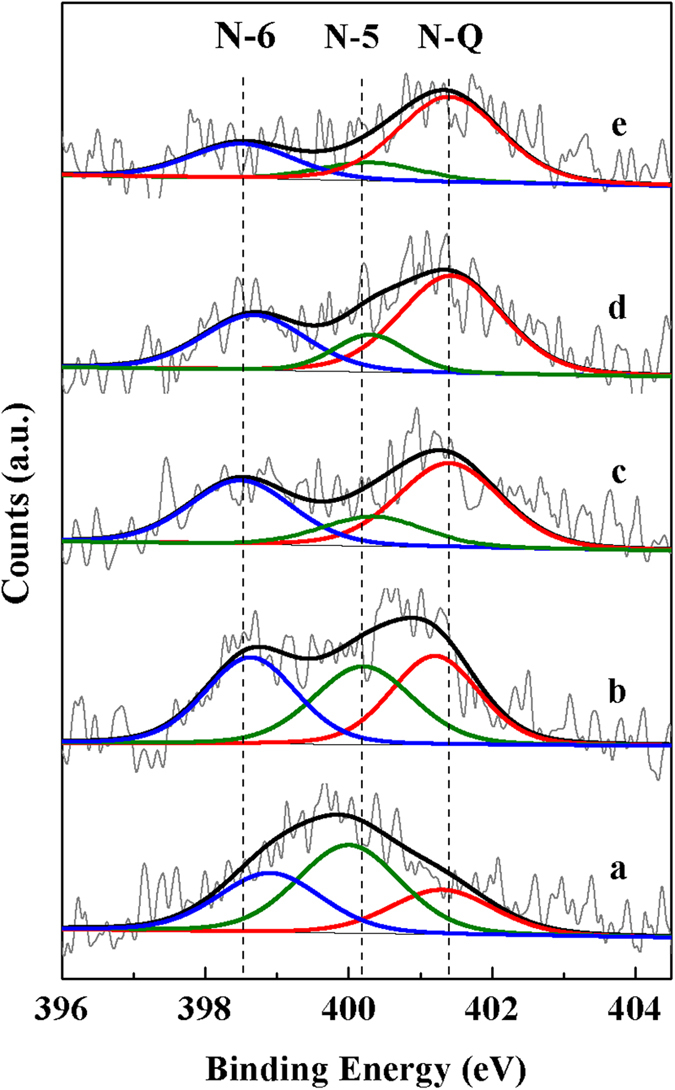
XPS (N1s) spectra of the FCC coked catalyst (**a**) original sample, (**b**) sample collected at the end of stage I, (**c**) sample collected at the end of stage II, (**d**) sample collected at the end of stage III, (**e**) sample collected at the end of stage IV.

Residual coke fractions were examined via X-ray photoelectron spectroscopy (XPS) to probe the change in surface speciation at different stages of the TPD procedure (Fig. 5). Initial coke exhibits a broad N1s peak centered at 399.8 eV (Fig. 5a). After being subjected to the initial TPD stage, the broad N1s peak split, and two shoulder peaks are generated at around 398.8 and 400.9 eV (Fig. 5b). During the TPD process, the shoulder peaks at low and high binding energy (BE) shifts toward lower and higher BE respectively, simultaneously, signal intensity of the shoulder peaks decrease with increased temperature and hold time.

XPS has been used to characterise model char in several works^8^,^25^,^29^. Kapteijn *et al.* reported that three types of chemical N species can be discriminated in char-type materials, i.e., pyridinic nitrogen (N-6), pyrrolic nitrogen (N-5) and quaternary nitrogen (Q-N)^29^. The pyridinic and pyrrolic nitrogen are located at the edge of the carbon structure with BE of 398.7 ± 0.2 eV and 400.3 ± 0.2 eV respectively, and Q-N represented by nitrogen substitutes for carbon in the “graphene” type structure exhibits strong interactions with acid sites and a higher BE of 401.4 ± 0.2 eV^29^. Reported references further demonstrated that the nature of FCC coke was similar to that of chars, hence, information of N functionality in model chars could be applied to characterise FCC coke^8^,^25^. In this study, N1s spectra are deconvoluted and the details are shown in Table 2. Three N species are differentiated in FCC coke, N species with BE values of 398.7, 400.2 and 401.2 eV are attributed to N-6, N-5 and Q-N, respectively. The fitting results (Table 2) indicate that the peak area of N-5 declines throughout TPD, N-6 however, exhibits an initial increase in peak area prior to a continual reduction. Q-N increases in peak area, except for the final stage. The results suggest that N-5 (composed of five-member structures) is the most unstable species, it decomposes via the disproportionation reaction, generating stable structures (N-6, Q-N) and contributing to the generation of NH_3_, HCN and NO (Fig. 5). This accords well with previous reports that pyrrolic nitrogen converts into pyridinic nitrogen and quaternary nitrogen with increased coking time^29^. N-6 is more stable than N-5 and primarily decomposes at higher temperature, however, Q-N is the most stable species and decomposes at the last TPD stage. This can be supported by the reported literature that Q-N in FCC coke has a strong interaction with the Brönsted acid sites of the catalyst^8^,^29^. Hence, it can be deduced from the XPS experiments that the formation of CO, CO_2_ and NO at the last two stages can be attributed to the reaction between N-6/Q-N and adjacent lattice oxygen atoms in the catalyst.

### Oxidation of the coked catalyst

**Figure 6:**
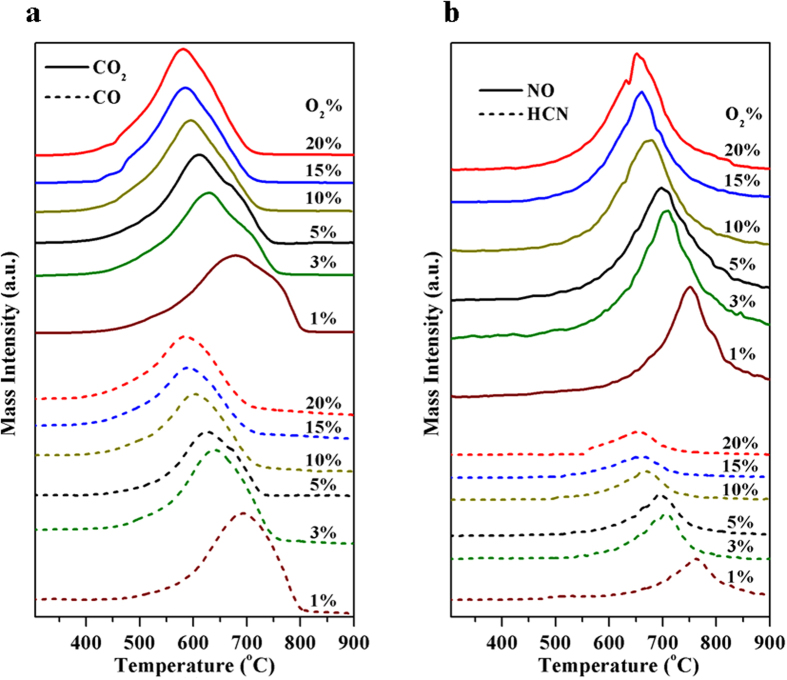
Gaseous species evolved during TPO at a uniform heating rate under various oxygen concentrations (**a**) carbon compounds, (**b**) nitrogen compounds; *P* = 0.25 MPa; 100 mL min^−1^ of *x*% O_2_/He, from 300 to 900 °C with heating rate of 10 °C min^−1^.

A series of TPO experiments were conducted under various oxygen concentrations (Fig. 6). TPO results obtained under 20% O_2_/He accord well with the TGA conducted in air (Fig. 1). Sample weight loss at 350–700 °C is due to oxidation of coke (particularly the oxidation of carbonaceous compounds) with generation of carbon and nitrogen oxides. Above 700 °C the sample weight hardly changes (Fig. 1), but NO is still detectable (Fig. 6b), this can be explained by the reaction of residual nitrogen containing coke species. The obtained TPO results state that with increasing oxygen concentration, the maximum generation temperatures of C/N oxides decrease (Fig. 6), and CO tends to be converted into CO_2_ with increased peak area ratio of CO_2_ to CO (Table S8). As reported previously, coke reaction contains two oxidative steps: 1) oxidation of C into CO, 2) CO oxidation into CO_2_^26^,^37^. In an oxygen rich environment the rate determining step for complete combustion is associated with the initial oxidation of carbon to CO followed by a relatively fast second oxidation^37^,^38^. In an oxygen lean atmosphere CO oxidation is less favourable and becomes the rate determining step for CO_2_ formation^37^. During the TPO process, HCN formation was observed (Fig. 6b), indicating that nitrogen compounds in FCC coke will be thermally decomposed into small molecules. Little NH_3_ was detectable during the TPO experiments, because the generation of NH_3_ needs more H radicals and this radical is easy to be oxidised at the presence of O_2_^39^,^40^.

**Figure 7:**
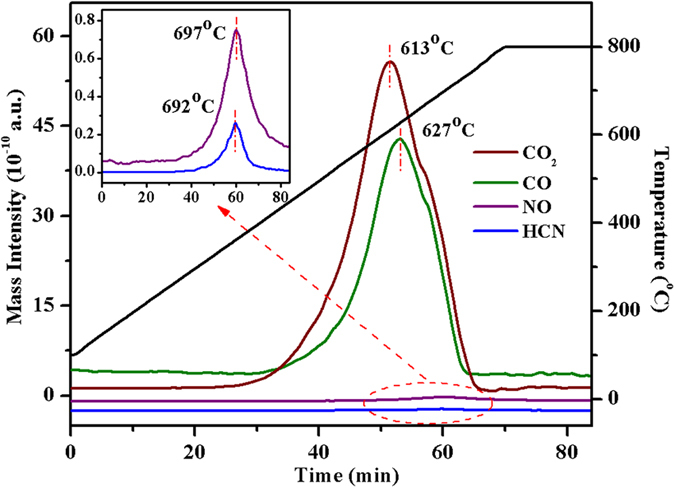
Gaseous species evolved during TPO (*P* = 0.25 MPa; 100 mL min^−1^ of 5%O_2_/He; from room temperature to 800 °C with 10 °C min^−1^ and held for 30 min at 800 °C).

A typical TPO experiment was conducted in 5% O_2_/He (Fig. 7), and the surface elements (C, N, Si and Al) of the samples were recorded by XPS (Table 3). Si and Al combined with oxygen constitute the main structure of the FCC catalyst, whereas, C and N are the main components of the FCC coke^4^. Table 3 shows that the content of C on the catalyst surface declines with increasing combustion temperature. The greatest drop occurs in the temperature range 500–650 °C with corresponding change of C percentage from 55.84% to 19.26%, this is consistent with the generation of CO_2_ and CO, and their maximum formation rates (613 and 627 °C respectively; Fig. 7). The greatest change of N occurs between 650 and 800 °C, and the maximum generation temperatures of HCN and NO are 692 and 697 °C, respectively (Fig. 7). Both TPO and XPS results indicate that N retention is observed during oxidation, this can also be explained by the preferential binding of basic nitrogen containing molecules on Brönsted acid sites, which are located primarily in the pores of the zeolite^4^,^8^. Thus C is more accessible to oxygen during TPO and tends to be oxidised preferentially.

**Figure 8:**
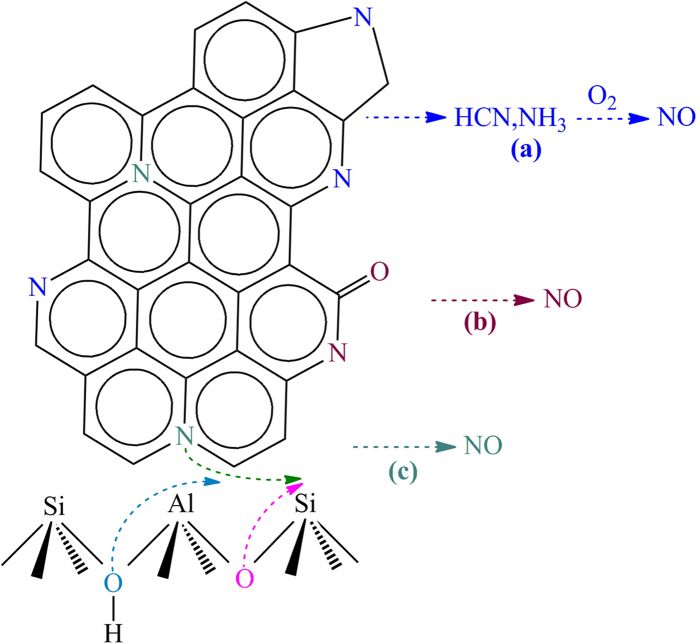
NO formation during the FCC regeneration.

Based on the investigations above, the formation of NO during FCC regeneration can be summarised (Fig. 8). Pyridinic and pyrrolic nitrogen, located at the edge of the carbon structure, are primarily decomposed into volatile intermediates such as HCN and NH_3_ (Fig. 8a)^8^,^24^. NO can be generated from the oxidation reaction between these intermediates and oxygen present in the regenerator^41^,^42^. NO can also be formed via the reaction between coke-N and oxygen atoms derived from the FCC coked catalyst. Pyridone, associated with carbon-oxygen functionalities, is able to generate NO through the reaction between coke-N and adjacent oxygen atoms (Fig. 8b). Q-N, exhibiting good thermal stability, tends to react with hydroxyl groups and lattice oxygen derived from the FCC catalyst (Fig. 8c).

In summary, the formation of N and C containing compounds over an industrial FCC coked catalyst during thermal regeneration was studied. Two coke types are identified via their thermal decomposition and the generated gases were monitored via online mass spectrometry. Oxidation products (NO, CO and CO_2_) are observed simultaneously under the O_2_-free atmosphere, indicating that oxygen arose from the coked catalyst. Three types of N species, i.e., pyrrolic N, pyridinic N and quaternary N are identified in the FCC coke. The former two are unstable and located at the outer layer of the coke, while quaternary N is stable and reacts only at high temperatures. Two routes of NO formation are proposed: (1) Pyrrolic N and pyridinic N are initially converted into volatile intermediates (primarily HCN and NH_3_) and these intermediates can readily be oxidised. (2) NO is formed via the direct reaction between coke-N and oxygen from the FCC coked catalyst.

## Method

### Sample preparation

The coked catalyst without CO combustion promoter was sampled from a typical FCC unit in Sinopec Zhongyuan refinery (Puyang, China), between the reaction and regeneration stages but after the stripping. In order to protect the coked catalyst from O_2_ and H_2_O in the air, the sample was extracted and stored in a helium atmosphere by referring to a Sinopec invention patent^43^. The basic physical properties and chemical composition of the FCC coked catalyst were measured and listed in Table S1. The coked catalyst was sieved to 140–230 mesh prior to use. SiO_2_, pretreated in a muffle furnace (Vulcan, 3–550) at 1000 °C for 6 hours, was used to dilute the sample to avoid sintering during the heating process.

### Temperature programmed decomposition/oxidation

The TPD and TPO experiments were performed in a micro-reactor system (Xianquan WFSM-3060) coupled with a mass spectrometer (MS, Hiden HPR 20) and a gas chromatograph (GC, Agilent 7890A, TCD detector, Agilent molsieve 5A column). About 720 mg sample was loaded in a quartz inner liner fixed in the stainless steel tubular reactor. The reactor was purged by a flow of 100 mL min^−1^ helium (99.999%) for 30 min to remove the air, and then heated to 300 °C and kept for 2 hours to get rid of bound water. Both TPD and TPO experiments were conducted under the pressure of 0.25 MPa in accordance with the actual industry process (0.24–0.30 MPa). The emitted gas was monitored by the on-line mass spectrometer and gas chromatograph.

The TPD experiments were conducted in a helium atmosphere at the flow of 100 mL min^−1^, the reactor was heated either directly up to 950 °C at the rate of 10 °C min^−1^ or according to a four-stage temperature program: heated from 300 to 750 °C at the rate of 25 °C min^−1^ (step I), held at 750 °C for 30 min (step II), heated from 750 to 950 °C at the rate of 20 °C min^−1^ (step III), and finally held at 950 °C for 30 min (step IV). In the TPO experiments, the reactor was heated either from 300 to 900 °C with the heating rate of 10 °C min^−1^ and under the atmosphere of *x*% O_2_/He (*x* = 1, 3, 5, 10, 15, 20) at the flow of 100 mL min^−1^, or from room temperature up to a certain temperature (350, 500, 650 and 800 °C, respectively) at the rate of 10 °C min^−1^ and in an atmosphere of 5% O_2_/He at the flow of 100 mL min^−1^.

### Characterisation

TGA was performed on a thermal analyser (NETZSCH STA449C). The coked catalysts were evaluated either in an air atmosphere or in a helium atmosphere (99.999% He), ramped up from 30 to 1100 °C at 10 °C min^−1^. After being submitted to TPD or TPO experiments, the samples were cooled to room temperature in the helium stream and separated from the mixed SiO_2_, and then they were adopted for NMR and XPS characterisations. The NMR experiments were performed with a Bruker Avance III HD 400 spectrometer, operating at ^13^C frequency of 100.62 MHz and ^1^H frequency of 399.87 MHz. Data were collected with a 4 mm MAS probe operating at a spinning rate of 6 KHz. Cross polarisation spectra of samples were obtained using a recycle delay of 1.0 s and ‘TOSS’ spinning sideband suppression. A minimum of 80 000 scans were collected on each sample. The XPS experiments were conducted on a Thermo Scientific ESCALAB 250Xi spectrometer, equipped with a monochromatic Al Kα X-ray source (*hv* = 1486.6 eV). The carbon 1 s peak at 284.68 eV was used as the internal standard for determining peak positions. Elemental contents were calculated from peak areas corrected by relative sensitivity factors through the Avantage software. In order to identify the types of N species from overlapping peaks, the non-symmetric N1s experimental envelopes were subjected to a de-convolution procedure, which employed a mix of Gaussian-Lorentzian (80/20) function and a Shirley baseline.

## Additional Information

**How to cite this article**: Shi, J. *et al.* Nitrogen Chemistry and Coke Transformation of FCC Coked Catalyst during the Regeneration Process. *Sci. Rep.* **6**, 27309; doi: 10.1038/srep27309 (2016).

## Supplementary Material

Supplementary Information

## Figures and Tables

**Table 1:** 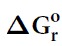
 (0.1 MPa) and Δ*G*_*r*_ (0.25 MPa) at different temperatures.

Item	298 K	300 K	400 K	500 K	600 K	700 K	800 K	900 K	1000 K
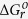 (kJ/mol)	−343.80	−343.60	−333.63	−323.56	−313.46	−303.38	−293.31	−283.28	−273.28
Δ*G*_*r*_ (kJ/mol)	−327.71	−327.40	−312.03	−296.56	−281.06	−265.58	−250.11	−234.68	−219.28

**Table 2:** N1s de-convoluted results of samples after four TPD stages.

Samples	Peak area			Peak area ratio		
	N-6	N-5	N-Q	N-6	N-5	N-Q
Original	240.2	358.4	181.6	0.31	0.46	0.23
After stage I	310.9	305.3	308.1	0.34	0.33	0.33
After stage II	259.8	120.7	342.4	0.36	0.17	0.47
After stage III	224.3	107.0	396.8	0.31	0.15	0.54
After stage IV	140.9	73.1	345.5	0.25	0.13	0.62

**Table 3:** Surface composition of samples at different temperatures of TPO.

NO.	Temperature (°C)	Composition (%)				Ratio of N/C (%)
		Al	Si	N	C	
1	original sample	4.58	4.42	0.81	63.24	1.28
2	350	5.06	4.42	0.79	61.30	1.29
3	500	5.48	5.86	0.75	55.84	1.34
4	650	13.22	12.42	0.68	19.26	3.53
5	800	15.20	14.06	0.54	10.08	5.36
