# Supplementary material for: Nitrogen Chemistry and Coke Transformation of FCC Coked Catalyst during the Regeneration Process
Source: Sci Rep. 2016 Jun 8;6:27309. doi: 10.1038/srep27309 (PMC4897648; doi:10.1038/srep27309)
Supplement: Supplementary Information [file srep27309-s1.pdf]

Supplementary Information

Nitrogen Chemistry and Coke Transformation of FCC Coked Catalyst during the Regeneration Process

Junjun Shi[1], Jianyu Guan[1], Dawei Guo[2*], Jiushun Zhang[2], Liam John France[1], Lefu Wang[1], Xuehui Li[1*]

[1] School of Chemistry and Chemical Engineering, Pulp & Paper Engineering State Key Laboratory of China, South China University of Technology, Guangzhou 510640, China

[2]Sinopec Research Institute of Petroleum Processing, Beijing 100083, China

[*] Corresponding author. Tel: 086 020 8711 4707, E-mail: cexhli@scut.edu.cn; Tel: 086 010 82368241, E-mail: guodawei.ripp@sinopec.com

Table S1 Physical properties and chemical composition of the FCC coked catalyst

Items	Values	Items	Values
Apparent density (g mL^{-1})	0.87	Na (*wt* %)	0.33
Specific area (m^2 g^{-1})	156	Sb (*wt* %)	0.11
Pore volume (mL g^{-1})	0.16	Ca (*wt* %)	0.18
RE_2O_3 (*wt* %)	1.82	Cu (*wt* %)	0.02
Al_2O_3 (*wt* %)	47.30	C (*wt* %)	1.59
SiO_2 (*wt* %)	41.91	N (ppm)	220
Fe (*wt* %)	0.48	S (ppm)	480
Ni (*wt* %)	0.29	Others (*wt* %)	5.65
V (*wt* %)	0.25		

Main component of rare earth oxides is Ce_2O_3.

Basic thermodynamic data. To figure out whether the reaction of NO + CO — $1/2N_2$ + CO_2 can be spontaneous or not, the Gibbs free energy of the reaction were calculated. According to the book of Physical Chemistry, basic thermodynamic data of NO, CO, CO_2 and N_2 were listed and shown in Table S2 and S3.[1]

Table S2 $\Delta H_f^o(298K)$, $\Delta S_f^o(298K)$ and $\Delta G_f^o(298K)$ of reactants and products

Item	$\Delta H_f^o(298K)$ (kJ/mol)	$\Delta S_f^o(298K)$ (J/mol·K)	$\Delta G_f^o(298K)$ (kJ/mol)
CO	-110.53	197.67	-137.17
NO	90.25	210.76	86.55
CO_2	-393.51	213.74	-394.36
N_2	0	191.61	0

Table S3 C_p of reactants and products at different temperatures

Item	C_p (J/mol·K)								
	298 K	300 K	400 K	500 K	600 K	700 K	800 K	900 K	1000 K
CO	29.14	29.16	29.33	29.79	30.46	31.17	31.88	32.59	33.18
NO	29.83	29.83	29.96	30.50	31.25	32.05	32.76	33.43	33.97
CO_2	37.11	37.20	41.30	44.60	47.32	49.54	51.42	52.97	54.27
N_2	29.12	29.12	29.25	29.58	30.11	30.76	31.43	32.10	32.70

Calculation of ΔH_r^o and ΔS_r^o at different temperatures. The Gibbs free energy can be calculated through the following formulas. Formula (1) represents the Gibbs function at nonstandard state, whereas formula (2) states that at standard state.

$$\Delta G_r(T) = \Delta G_r^o(T) + RT\ln K \qquad \text{Eq. (A.1)}$$

$$\Delta G_r^o(T) = \Delta H_r^o(T) - T\Delta S_r^o(T) \qquad \text{Eq. (A.2)}$$

$$K = \frac{[p(N_2)/P^o]^{0.5}[p(CO_2)/P^o]}{[p(CO)/P^o][p(NO)/P^o]} \qquad \text{Eq. (A.3)}$$

First of all ΔH_r^o and ΔS_r^o at different temperatures were calculated. The relationship between $\Delta H_r^o / \Delta S_r^o$ and temperature can be expressed as formula (4) and (5),

$$d\Delta H_r^o / dT = \Delta C_p \qquad \text{Eq. (A.4)}$$

$$d\Delta S_r^o / dT = \Delta C_p / T \qquad \text{Eq. (A.5)}$$

that is,

$$\Delta H_r^o(T_j) = \sum v_i \Delta H_f^o(T_{j-1}) + \sum v_i \bar{C}_{pi} \times (T_j - T_{j-1}) \qquad \text{Eq. (A.6)}$$

$$\Delta S_r^o(T_j) = \sum v_i \Delta S_f^o(T_{j-1}) + \sum v_i \bar{C}_{pi} \times \ln(T_j / T_{j-1}) \qquad \text{Eq. (A.7)}$$

$$\bar{C}_{pi} = \frac{C_{pi}(T_{j-1}) + C_{pi}(T_j)}{2} \qquad \text{Eq. (A.8)}$$

Thus the ΔH_r^o and ΔS_r^o at different temperatures can be calculated through formula (6), (7) and (8), based on the basic thermodynamic data of NO, CO, CO_2 and N_2 (Table S2 and S3). The calculation results were listed in Table S4.

Table S4 ΔH_r^o and ΔS_r^o of reactants and products at different temperatures

Items	298 K	300 K	400 K	500 K	600 K	700 K	800 K	900 K	1000 K
ΔH_r^o (kJ/mol)	-373.27	-373.28	-373.81	-374.03	-374.04	-373.92	-373.71	-373.44	-373.11
ΔS_r^o (J/K mol)	-98.89	-98.94	-100.46	-100.94	-100.96	-100.78	-100.50	-100.17	-99.83

Table S4 shows that effects of temperature on both ΔH_r^o and ΔS_r^o are very small, this is in agreement with the fact that variation of temperature has small effect on the C_p of gas. According to the results presented in Table S4 the ΔG_r^o at different temperatures can be calculated through the formula (2). And the calculation results are listed in Table S5.

Table S5 ΔG_r^o at different temperatures

Items	298 K	300 K	400 K	500 K	600 K	700 K	800 K	900 K	1000 K
ΔG_r^o (kJ/mol)	-343.80	-343.60	-333.63	-323.56	-313.46	-303.38	-293.31	-283.28	-273.28

Gibbs free energy at the nonstandard state (0.25 Mpa) is also calculated. In this study, the concentrations of CO、NO、N_2 and CO_2 are extremely low (ppm). Thus K can be calculated as following.

$$\frac{[(10^{-6} \times 0.25Mpa)/(0.1Mpa)]^{0.5}[(10^{-6} \times 0.25Mpa)/(0.1Mpa]}{[(10^{-6} \times 0.25Mpa)/(0.1Mpa)][(10^{-6} \times 0.25Mpa)/(0.1Mpa)]} = 632.45$$

Hence $\ln K = 6.45$, and the Gibbs free energy at the nonstandard state can be simplified as

$$\Delta G_r(T) = \Delta G_r^o(T) + 0.054T \qquad \text{Eq. (A.9)}$$

The ΔG_r at 0.25 Mpa can be calculated through (9) and the results are listed in Table S6.

Table S6 ΔG_r at different temperatures (0.25 Mpa)

Items	298 K	300 K	400 K	500 K	600 K	700 K	800 K	900 K	1000 K
ΔG_r (kJ/mol)	-327.71	-327.40	-312.03	-296.56	-281.06	-265.58	-250.11	-234.68	-219.28

The Signal-noise-ratio of the XPS data. The Signal-noise-ratios of the XPS data are calculated as following.[2]

$$P_{signal} = \text{Fitting data} - \text{Baseline}$$

$$P_{noise} = \text{Fitting data} - \text{original data}$$

$$\delta^2_{signal} = \sum (P_{signal} - \bar{P}_{signal})^2$$

$$\delta^2_{noise} = \sum (P_{noise} - \bar{P}_{noise})^2$$

$$SNR = \frac{\delta^2_{signal}}{\delta^2_{noise}}$$

$$SNR_{dB} = 10\log_{10}(SNR)$$

Table S7 Signal-noise-ratio of the XPS data

Item	SNR	SNR_{dB}
Sample a	4.02	6.04
Sample b	4.71	6.73
Sample c	2.71	4.33
Sample d	2.61	4.16
Sample e	1.97	2.94

Engineers consider a SNR of 2/3 (dB) to be the boundary between low and high SNRs.[2] Table S7 shows that the fitting results of the XPS experiments is acceptable, except for Sample e with the SNR less than 3. We think that it is acceptable considering such low N content in the sample.

Influence of O_2 concentration on coke oxidation. A series of TPO experiments were conducted under various oxygen concentrations to understand the influence of O_2 concentration on FCC coke oxidation. Mass signals of TPO products are fitted with the Least Squares Method and the obtained results are listed in Table S8.

Table S8 Influence of O_2 concentration on coke oxidation

Oxygen Concentration (%)	Peak temperature (^oC)				Peak area (10^{-7} a.u.)				Peak area ratio	
	CO_2	CO	NO	HCN	CO_2	CO	NO[a]	HCN[a]	CO_2/CO	NO/HCN
1	681	695	749	759	8.8	8.59	6.18	2.37	1.02	2.61
3	633	639	713	701	7.95	7.39	9.06	2.78	1.08	3.26
5	613	627	697	692	8.05	6.25	8.76	2.54	1.29	3.45
10	592	606	681	669	8.12	5.93	12.95	2.68	1.37	4.83
15	581	589	661	661	8.23	5.56	14.39	2.73	1.48	5.27
20	582	582	647	647	8.97	5.86	14.59	2.51	1.53	5.81

a) Signals of NO and HCN have been magnified 100 times.

Supplementary Reference

1. Fu, X. C., Sheng, W. X., Yao, T. Y. & Hou. W. H. *Physical Chemistry5th edn*, Ch. Appendix, 483-485 (High Education Press, 2010).
2. D.H. Johnson. Signal-to-noise ratio, Scholarpedia. 1(12):2088 (2006).
